# Mid-Regional Proadrenomedullin (MR-proADM) and Microcirculation in Monitoring Organ Dysfunction of Critical Care Patients With Infection: A Prospective Observational Pilot Study

**DOI:** 10.3389/fmed.2021.680244

**Published:** 2021-11-30

**Authors:** Roberta Domizi, Elisa Damiani, Claudia Scorcella, Andrea Carsetti, Paolo Giaccaglia, Erika Casarotta, Jonathan Montomoli, Vincenzo Gabbanelli, Marina Brugia, Marco Moretti, Erica Adrario, Abele Donati

**Affiliations:** ^1^Anesthesia and Intensive Care Unit, Azienda Ospedaliera Universitaria Ospedali Riuniti, Ancona, Italy; ^2^Department of Biomedical Sciences and Public Health, Università Politecnica delle Marche, Ancona, Italy; ^3^Laboratory Medicine, Azienda Ospedaliera Universitaria Ospedali Riuniti Ancona, Ancona, Italy

**Keywords:** mid-regional proadrenomedullin, microcirculation, sepsis, septic shock, infection, organ failure

## Abstract

**Introduction:** Microvascular alterations are involved in the development of organ injury in critical care patients. Mid-regional proadrenomedullin (MR-proADM) may predict organ damage and its evolution. The main objective of this study was to assess the correlation between MR-proADM and microvascular flow index (MFI) in a small cohort of 20 adult critical care patients diagnosed with infection, sepsis, or septic shock. Further objectives were to evaluate the correlation between the clearance of MR-proADM and the variables of microcirculation and between MR-proADM and the Sequential Organ Failure Assessment (SOFA) score.

**Materials and Methods:** This is a prospective observational pilot study. Inclusion criteria: consecutive adult patients admitted to intensive care unit (ICU) for or with infection-related illness. Daily measurement of MR-proADM and calculation of the SOFA score from admission in ICU to day 5. Repeated evaluations of sublingual microcirculation, collection of clinical data, and laboratory tests.

**Results:** Primary outcome: MR-proADM was not significantly correlated to the MFI at admission in ICU. A clearance of MR-proADM of 20% or more in the first 24 h was related to the improvement of the MFIs and MFIt [percentual variation of the MFIs + 12.35 (6.01–14.59)% vs. +2.23 (−4.45–6.01)%, *p* = 0.005; MFIt +9.09 (4.53–16.26)% vs. −1.43 (−4.36–3.12)%, *p* = 0.002].

**Conclusion:** This study did not support a direct correlation of MR-proADM with the MFI at admission in ICU; however, it showed a good correlation between the clearance of MR-proADM, MFI, and other microvascular variables. This study also supported the prognostic value of the marker. Adequately powered studies should be performed to confirm the findings.

## Introduction

Sepsis is a life-threatening syndrome characterized by a widespread tissue and microvascular injury (1, 2).

Organ failure is one of the main challenges of septic patients and hemodynamic optimization is a cornerstone of adequate organ perfusion for prevention and treatment of organ dysfunction (3).

However, organ sufferance may occur even after restoration of systemic hemodynamics. The mechanisms underlying this phenomenon are multifactorial and not completely clear, but there is increasing evidence that alterations of the microvascular blood flow are strongly implicated. Sepsis affects endothelial cell function; it determines endothelial barrier disruption and leakage and it leads to microcirculatory alterations that directly contribute to organ dysfunction (4–7).

Adrenomedullin (ADM) is an endogenous peptide hormone of 52 amino acids synthesized widely through tissues (including bone, adrenal cortex, kidney, lung, blood vessels, and heart). ADM is biologically active and its effects include vasodilator, positive inotropic, diuretic, natriuretic, and bronchodilator actions; ADM is also an inhibitor for the secretion of insulin, aldosterone, and adrenocorticotropic hormone (8, 9). Previous studies showed that ADM increases in inflammatory diseases, including sepsis and septic shock, in order to stabilize the microcirculation and to protect against endothelial hyperpermeability (10–14); they also suggested that the variation in plasmatic levels of ADM may act as a marker of severity of the endothelial damage (15, 16).

Mid-regional proADM (MR-proADM) is a fragment of ADM with no known function. It is produced in ratio of 1:1 to ADM. Its half-life is numerable in hours and it proportionally reflects the activity of ADM (8).

Mid-regional proADM was already described as biomarker in community-acquired pneumonia and it has been proposed as a prognostic marker with potential clinical role in sepsis (17, 18). In the study of Valenzuela-Sánchez et al. (19), MR-proADM showed good correlation with organ dysfunction related to infection and to mortality in critical care patients. The authors evaluated the clearance of MR-proADM during the first days of intensive care unit (ICU) admission and they demonstrated an enhanced clearance of MR-proADM in survivor patients.

In our small-scale preliminary study, we aimed to investigate if MR-proADM, as biomarker of organ failure and of endothelial damage, could be correlated with microvascular alterations in critical care patients admitted in ICU for or with different degrees of infection-related illness.

## Materials and Methods

### Population, Enrolment, and Data Collection

This is a prospective observational pilot study performed in the 14-bed General and Traumatic ICU of Azienda Ospedaliera Universitaria Ospedali Riuniti of Ancona (Italy).

Inclusion criteria: 20 adult (age equal or superior to 18 years old) critically ill patients, consecutively admitted in ICU for or with different degrees of infection-related illness (infection, sepsis, and septic shock), with a length of stay (LOS) in ICU inferior to 24 h before the enrollment in this study. Infection, sepsis, and septic shock were determined according to the Third International Consensus Definitions for Sepsis and Septic Shock (20).

Exclusion Criteria to enrollment were: age inferior to 18 years old, LOS in hospital longer than 48 h, conditions that prevented adequate monitoring of sublingual microcirculation, end-of-life care, and refusal to consent.

The primary objective of this study was to study a correlation between plasma levels of MR-proADM and the microcircular flow index (MFI) at admission in ICU. Sample size was calculated on the primary endpoint. It was also purpose of the study to examine the relationship between MR-proADM in the 5-day period of observation and other microvascular variables [total vessel density (TVD), De Backer score, perfused vessel density (PVD), and proportion of perfused vessels (PPV)] and to verify the association of MR-proADM with the Sequential Organ Failure Assessment (SOFA) score (as marker of organ dysfunction) and between the SOFA score and the microvascular indices. We predetermined to calculate the clearance of MR-proADM and to evaluate the relation between the first 24 h clearance and the evolution of microvascular parameters.

The study was articulated in 5 days of monitoring (day 1 to day 5) from admission in ICU.

Plasmatic levels of MR-proADM were dosed for all the timepoints. The Simplified Acute Physiology Score (SAPS) II and the Acute Physiologic Assessment and Chronic Health Evaluation Classification System II (APACHE II) scores were calculated at admission in ICU. The SOFA score was evaluated at admission (day 1) and daily to day 5.

Anthropometric and demographic data were collected at baseline including age, sex, weight, and height of the patients.

Microbiological parameters were assessed to categorize patients [infection, sepsis, or septic shock; source of infection; and presence of multidrug resistance (MDR)].

For each of the five timepoints, we recorded clinical, hemodynamic, and laboratory parameters [systolic, diastolic, and mean arterial pressure; heart rate; cardiac output where available; respiratory parameters and mechanical ventilation; venous and arterial blood gas variables; vasoactive therapy; main parameters for renal, hepatic, and hematological function; and procalcitonin (PCT)].

At day 1 (<24 h from ICU admission), day 2 (24 h after the first assessment), and day 5, the sublingual microcirculation was assessed by using incident dark field (IDF) technology.

### Microvascular Assessment

Sublingual microcirculation was registered by using a high-resolution video microscopy camera (CytoCam, Braedius Medical BV, Huizen, Netherlands, UK) with IDF technology and the microcirculatory parameters were, then, derived offline with the Automated Vascular Analysis (AVA) software (version 3.2; Microvision Medical, Amsterdam, Netherlands, UK).

Microvascular assessment and analysis were performed by experienced operators and in compliance with the “second consensus on the assessment of sublingual microcirculation in critically ill patients” and “the microcirculation image quality score” (21, 22).

The MFI, TVD, PVD, and PPV were calculated for both the small-size vessels and total vessels in all the videos analyzed. The De Backer score was analyzed for total vessels.

The MFI is a semi-quantitative measure of perfusion quality; it is calculated by dividing the image into four quadrants in which the observer reports the predominant type of flow by using an ordinal scale (0 for absent flow, 1 for intermittent flow, 2 for sluggish flow, and 3 for normal flow).The average of the four quadrants is the final MFI.

Total vessel density and De Backer score are indices of vessel density. The first is the total length of vessels divided by the total surface of the analyzed area, while the second one is calculated as the number of vessels crossing horizontal and the vertical arbitrary grid lines divided by the total length of the lines. PPV is the percentual number of perfused vessels divided by the total number of vessels; PVD is derived by multiplying vessel density by the PPV and reflects the functional vessel density.

### Measurement of MR-proADM

Arterial blood samples were collected and immediately centrifuged. Plasma samples were, then, stored at −80°C for subsequent measurement of MR-proADM.

Plasmatic levels of MR-proADM were measured by using Time Resolved Amplified Cryptate Emission (TRACE) technology with Thermo Scientific™ B·R·A·H·M·S™ KRYPTOR Compact PLUS (Dasit). The reference limit for this method was 0.55 nmol/l.

### Ethics

In compliance with national applicable laws, informed consent was obtained from the subject before inclusion by signing the appropriate informed consent paperwork. Patients temporarily unable to consent were included in the study with deferred subject consent in a later phase and written informative for the next of kin. The study protocol was approved by the Local Ethics Committee [Comitato Etico Regione Marche (CERM); protocol number 212639, NCT03931967] and it conformed to the principles of Helsinki declaration (last revision, Edinburgh 2000).

### Sample Size Calculation

Sample size calculation was calculated on the basis of the primary endpoint of the study (correlation between plasma levels of MR-proADM and the MFI at admission in ICU-T1): 19 patients were shown to be sufficient to detect a statistically significant correlation coefficient (higher than 0.6) with a power of 80% and an alpha error of 0.05.

### Statistical Analysis

Statistical analysis was performed by using IBM SPSS statistic software (version 17.0) (IBM Corporation, New York, USA).

According to the distribution of the main variables (assessed with the Kolmogorov–Smirnov test) and to the limited size of the sample, non-parametric statistics predominated. Data are presented as median and interquartile ranges (IQRs) for continuous variables and number and percentage for discrete variables.

The Spearman's rank correlation coefficient was used to summarize the strength and direction (negative or positive) of the relationship between MR-proADM and the MFI as primary outcome measure with further parameters of microcirculation and with the severity scales. The non-parametric Mann–Whitney *U* test was used for comparisons between independent samples. The Friedman test with the Dunn's *post-hoc* pairwise comparison was used for repeated measures of the same variable. In order to take into account the factor “time” in the comparison between groups, the two-way ANOVA for repeated measures was also performed (after normalization of the data through Box-Cox transformation) for the parameter of microcirculation with the Sidack's *post-hoc* test. The area under the receiver operating characteristic (ROC) curve was calculated to sample the ability of MR-proADM to discriminate the severity of patients.

Differences were considered significant at *p* < 0.05.

## Results

### Descriptive of the Sample

From November 2018 to June 2019, a total of 29 patients were screened for the study and 20 of them were enrolled after obtaining the informed consent. A total of 9 patients were not enrolled for exclusion criteria.

Patients were predominantly males (65%) with a median age of 70 (51–74) years. At admission in ICU, the SAPS II score was 52.5 (35.50–75.05), the APACHE II score was 19.5 (12.25–30.00), and the SOFA score was 11 (8–14). The SOFA score at admission corresponded to SOFA score at time of enrollment, as all the patients were enrolled in the first 24 h of ICU stay (Table 1).

**Table 1:** Descriptive of the study population.

Age, years	70 [51–74]
Males	13 (65)
SAPS II score, AU	52.50 [35.50–75.05]
APACHE II score, AU	19.50 [12.25–30.00]
SOFA score, AU	11 (8–14)
Source of infection	
Respiratory	13 (65)
Abdominal	3 (15)
Genito-urinary	2 (10)
CNS	2 (10)
Septic shock	10 (50)
Sepsis	5 (25)
Infection without sepsis	5 (25)
PCT at admission, ng/ml	6.42 [1.01–24.08]
WBC at admission, cell ^*^ 10^3^/mm^3^	9.71 [7.7–13.06]
LOS in ICU, days	12.50 [9.00–16.75]
Mortality	3 (15)

*Median (IQR), n (%), AU*.

*IQR, interquartile range; SAPS II, Simplified Acute Physiology II; APACHE II, acute physiology and chronic health evaluation II; SOFA, Sequential Organ Failure Assessment; PCT, procalcitonin; WBC, white blood cell; LOS, length of stay*.

Half of the 20 patients were in septic shock at recruitment and 5 of 20 patients were septic. The origin of infection was respiratory in the vast majority of them (65%). Of 13 patients with low respiratory tract infections, five patients were diagnosed with type 1 influenza virus. Mortality rate was 15% and all the non-survivors were in septic shock. The LOS in ICU was 12.50 (9.00–16.75) days, but 11 of 20 patients were transferred to other ICUs for further treatments (Table 1). Median LOS for patients who did not survive was 21 (9–21) days.

Mid-regional proADM was 3.42 (1.73–4.17) nmol/l at day 1, 3.02 (1.55–3.73) nmol/l at day 2, 2.05 (1.35–3.59) nmol/l at day 3, 1.8 (1.34–3.10) nmol/l at day 4, and 1.62 (1.27–3.20) nmol/l at day 5.

We calculated the clearance of MR-proADM (daily clearance in percentage) during the 5 days of recruitment: the median clearance of MR-proADM was 11.44 (−12.29–23.90)% at day 2, 10.79 (0.16–26.94)% at day 3, 1.80 (−6.82–16.38)% at day 4, and 8.82 (−2.35–20.61)% at day 5.

In Table 2 we report the median values of MR-proADM and of PCT at admission in ICU (day 1 of enrollment) according to the diagnosis (infection, sepsis, and septic shock). No significant difference was evidenced for MR-proADM and PCT in the comparison between infected, septic, and septic shock patients.

**Table 2:** Day 1 plasmatic values of MR-proADM and procalcitonin (PCT).

	**Infection**	**Sepsis**	**Septic shock**	**p value**
MR-proADM, nmol/l	1.44 [0.94–1.84]	2.16 [1.35–4.00]	3.99 [3.58–7.04]	ns
PCT, ng/ml	1.20 [0.52-6.93]	0.8 [0.59-3.73]	25.95 [1.79-86.62]	ns

*Median (IQR)*.

*MR-proADM, mid-regional proadrenomedullin*.

The median values of MR-proADM at admission were higher in septic shock confronted to sepsis and of sepsis confronted to infection; these differences are not statistically significant. PCT did not show a linear increase in the three subgroups and resulted in very wide IQRs. MR-proADM and PCT showed a weak linear correlation in the population, when evaluated at T1 (Spearman's rank correlation coefficient + 0.672, *p* = 0.001). The correlation was more solid in the subgroup of patients with septic shock (Spearman's rank correlation coefficient + 0.758, *p* = 0.011).

### Mid-Regional proADM and Microvascular Flow Index

Table 3 presents median values of the parameters of microcirculation in the general population at the three timepoints.

**Table 3:** Descriptive of microvascular parameters at day 1, day 2, and day 5.

	**Day 1**	**Day 2**	**Day 5**	***p* value**
MFIs, AU	2.83 [2.67–2.83]	2.92 [2.75–2.00]	2.75 [2.58–2.92]	ns
MFIt, AU	2.87 [2.75–2.92]	2.92 [2.86–3.00]	2.83 [2.75–2.96]	ns
TVDs, mm/mm^2^	21.20 [17.07–23.85]	18.98 [16.99–23.11]	20.31 [18.29–23.17]	ns
TVDt, mm/mm^2^	21.87 [18.21–25.38]	19,60 [18.33–23.68]	20.94 [19.15–24.10]	ns
PVDs, mm/mm^2^	20.10 [16.55–23.04]	18.88 [16.88–23.00]	18.70 [17.49–22.72]	ns
PVDt, mm/mm^2^	20.85 [17.70–24.86]	19.41 [18.21–23.57]	20.72 [18.83–23.52]	ns
PPVs, %	97.01 [95.47–98.01]	98.57 [96.26–99.39]	96.86 [95.14–98.71]^*^	0.023
PPVt, %	97.04 [96.53–98.35]	98.65 [96.61–99.41]	97.11 [95.05–98.59]	ns
De Backer score, 1/mm	12.99 [10.29–13.96]	11.28 [10.18–13.27]	11.77 [11.14–13.83]	ns

*Median (IQR) [Friedman test with Dunn's post-hoc test*.

* *p < 0.05 in the comparison t_(5)_ on t_(2)_]*.

*MFIs, microvascular flow index of small vessels; MFIt, microvascular flow index of total vessels; TVDs, total vessel density; TVDt, total vessel density of total vessels; PVDs, perfused vessel density; PVDt, perfused vessel density of total vessels; PPVs, proportion of perfused small vessels; PPVt, proportion of perfused vessels for total vessels*.

The MFI of small vessels (MFIs) at admission in ICU was lower in the subgroup of septic shock patients [2.72 (2.5–2.85)], but not statistically different from that of septic [2.83 (2.83–2.96)] and infected [2.83 (2.58–2.87)] patients. Similar results were evident for MFI of total vessels (MFIt).

Both the MFIs and MFIt at admission in ICU were not correlated to MR-proADM (the Spearman's rank correlation coefficient not statistically significant) in the general population nor in the three subgroups of patients (infected, septic, and shocked).

We determined an arbitrary cutoff of 20% of the clearance of MR-proADM in the first 24 h of recruitment by dividing the patients in two groups: patients who showed a clearance of MR-proADM higher and equal-to-lower than 20%.

**Figure 1:**
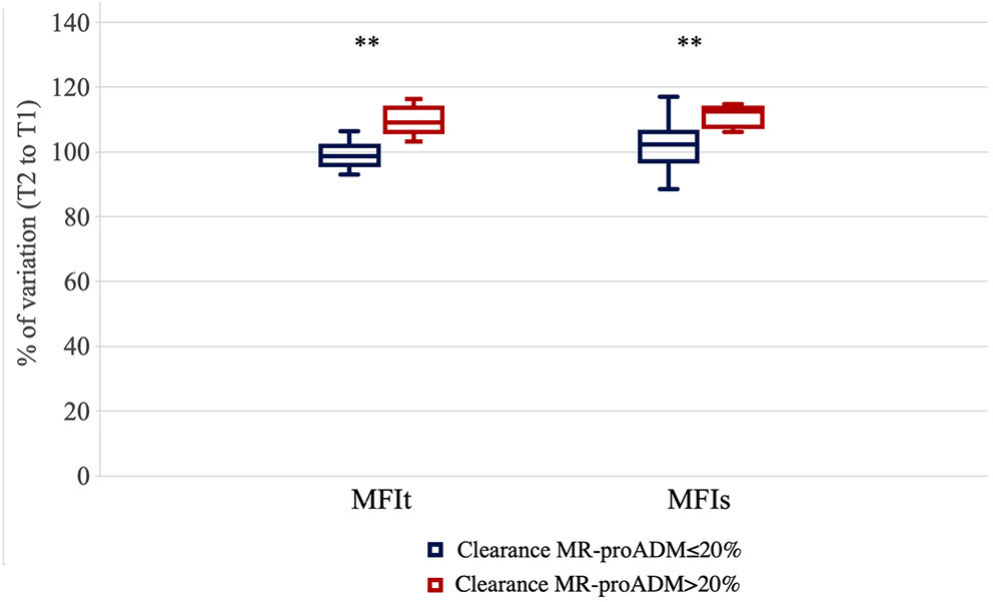
Percentage of variation for microvascular flow index of total vessels (MFIt) and MFI of small vessels (MFIs) in the first 24 h of evaluation in the two groups of patients [clearance of mid-regional proadrenomedullin (MR-proADM) inferior-to-equal or higher than 20%]. ^**^p < 0.01.

We measured the percentage of variation of the MFI of small and total vessels in the same time frame. The MFI improved significantly more in patients that showed a clearance of MR-proADM > 20% compared to those patients where the clearance of MR-proADM was ≤ 20% [the Mann–Whitney *U* test, percentual variation of the MFIs +12.35 (6.01–14.59)% vs. +2.23 (−4.45–6.01)%, *p* = 0.005; percentual variation of MFIt + 9.09 (4.53–16.26)% vs. −1.43 (−4.36–3.12)% *p* = 0.002] (Figure 1).

Patients with reduced clearance of MR-proADM showed lower clearance also at T5 (clearance T5 to T2 19.2 vs. 24.3%) and deterioration in the MFIs [negative variation of −6.1% (−10.5–0) vs. 0% (−6.33–(+3.1); *p* = 0.017]. The trend was similar for MFIt, but not statistically significant (*p* = 0.06).

**Figure 2:**
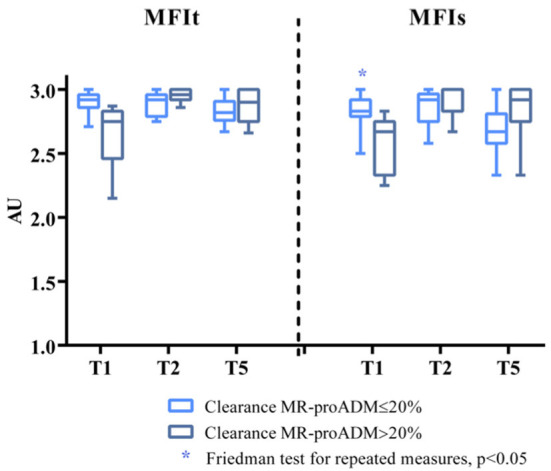
MFIs and MFIt at T1, T2, and T5 in the two groups of patients (clearance of MR-proADM inferior-to-equal or higher than 20%). The Friedman test for repeated measures statistically significant for MFIs in the group of patients with clearance of MR-proADM ≤ 20%. ^*^p < 0.05.

The Friedman test for repeated measures was statistically significant for the MFIs in the group of patients with clearance of MR-proADM ≤ 20% (*p* = 0.035, Dunn's *post-hoc* test not significant; *p* = 0.057 for patients with clearance of MR-proADM > 20%) (Figure 2).

**Figure 3:**
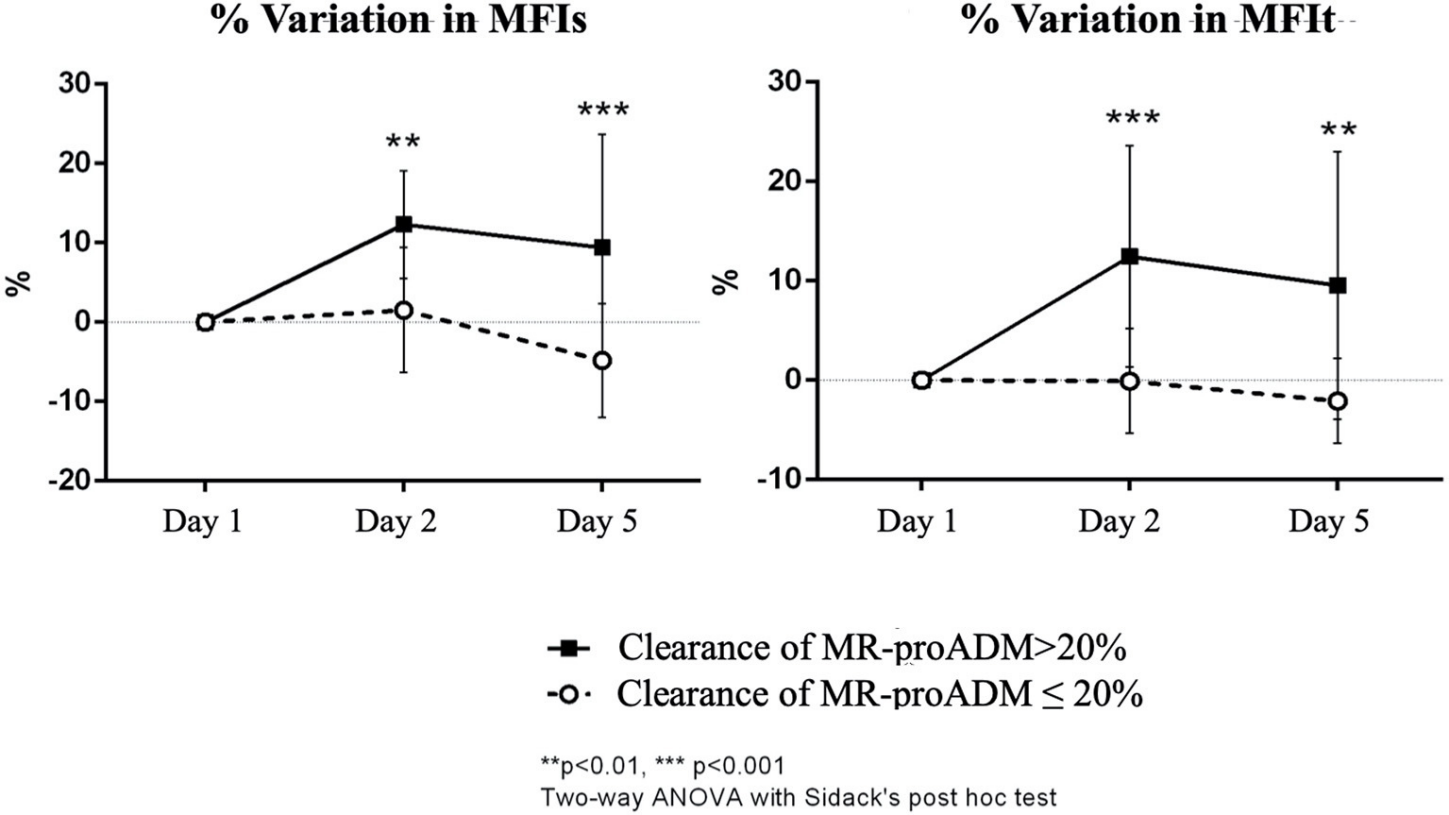
Percentual variation of MFIs and MFIt from baseline in the two groups of patients (clearance of MR-proADM inferior-to-equal or higher than 20%). The two-way ANOVA with the Sidack's *post-hoc* test. ^**^p < 0.01. ^***^p < 0.001.

The two-way ANOVA for repeated measures, performed after normalization of the data through Box-Cox transformation, showed for the MFIs a significant interaction between time and group (*p* = 0.015), but no significant effect of time (*p* = 0.611) or group (*p* = 0.836) *per se*. The Sidack's multiple comparisons test revealed a significant difference at baseline between the two groups (*p* = 0.043) and no other differences at the other time points [*t*_(2)_: *p* = 0.848; t_(5)_: *p* = 0.419]. Moreover, there was a significant increase in the MFIs at day 5 only in the group of patients with clearance of MR-proADM > 20% (*p* = 0.045 vs. baseline). The two-way ANOVA was also performed on the percentage of variation of the MFIs from baseline and it showed a significant interaction between time and group (*p* = 0.005) and a significant effect of both the time (*p* = 0.008) and group (*p* = 0.004). The Sidack's multiple comparisons test revealed a significant difference between the two groups at 24 h (*p* = 0.009) and at day 5 (*p* < 0.001). A significant increase in the MFIs was found only in the group of patients with a clearance of MR-proADM > 20% either at 24 h (*p* = 0.002 vs. baseline) and 5 days (*p* = 0.017 vs. baseline), while the MFIs did not significantly change over time in patients with a clearance of MR-proADM ≤ 20% (Figure 3).

For MFIt, the two-way ANOVA test showed a significant interaction between time and group (*p* = 0.004). No significant effect of time (*p* = 0.561) or group (*p* = 0.937) was noticed. There was a significant difference at baseline between the two groups (*p* = 0.029; Sidack's multiple comparisons test) and no other differences at the other time points [t_(2)_: *p* = 0.273; t_(5)_: *p* = 0.780]. Moreover, there was a significant increase in MFIt at day 2 only in the group of patients with clearance of MR-proADM > 20% (*p* = 0.018 vs. baseline). The analysis performed on percentage of variation of MFIt from baseline showed a significant interaction between time and group (*p* = 0.001) and a significant effect of both the time (*p* = 0.003) and group (*p* = 0.006). The difference between the two groups was significant at 24 h (*p* < 0.001) and at day 5 (*p* = 0.002) and a significant increase in MFIt was found only in the group of patients with clearance of MR-proADM > 20% either at 24 h (*p* < 0.001 vs. baseline) and 5 days (*p* = 0.002 vs. baseline) (Figure 3).

### Mid-Regional proADM and Other Parameters of Sublingual Microcirculation

Mid-regional proADM was not correlated to the parameters of sublingual microcirculation at admission in ICU. The proportion of perfused small and total vessels were weakly correlated to MR-proADM at day 2 (Spearman's rho correlation coefficient for PPVs−0.648, *p* = 0.002; for PPVt−0.578, *p* = 0.008).

The Mann–Whitney *U* test showed a difference in the percentual variation of PVDs and PVDt day 2 to day 1 between patients with a clearance of MR-proADM > 20% and ≤ 20%, respectively; the percentual variation of PVDs was +10.51 [−9.28–(+)15.09]% vs. −9.93 [−19.32–(–)3.94]%; *p* = 0.024] and the percentage of variation of PVDt was 6.94 [−8.98–(+)23.12]% vs. −9.65 [−16.15–(–)4.35]%; *p* = 0.024].

The difference between the two groups was shown also in the interval day 5 to day 1 [percentage of variation of PVDs +20.19 (2.26–33.57)% vs. −14.24 [−27.05–(+)0.17]%; *p* = 0.005] [PVDt + 15.43 (0.68–32.67)% vs. −11.27 [−23.23–(+)3.42]%; *p* = 0.01].

The Friedman test for repeated measures was statistically significant for PVDs and PPVs in the group that showed a reduced clearance of MR-proADM (*p* = 0.039 for PVDs, the Friedman test for repeated measures, the Dunn's *post-hoc* test significant in the comparison T5 to T1, *p* = 0.043; *p* = 0.027 for PPVs, the Dunn's *post-hoc* test significant in the comparison T5 to T2, *p* = 0.024) (Supplementary Figure 1).

**Figure 4:**
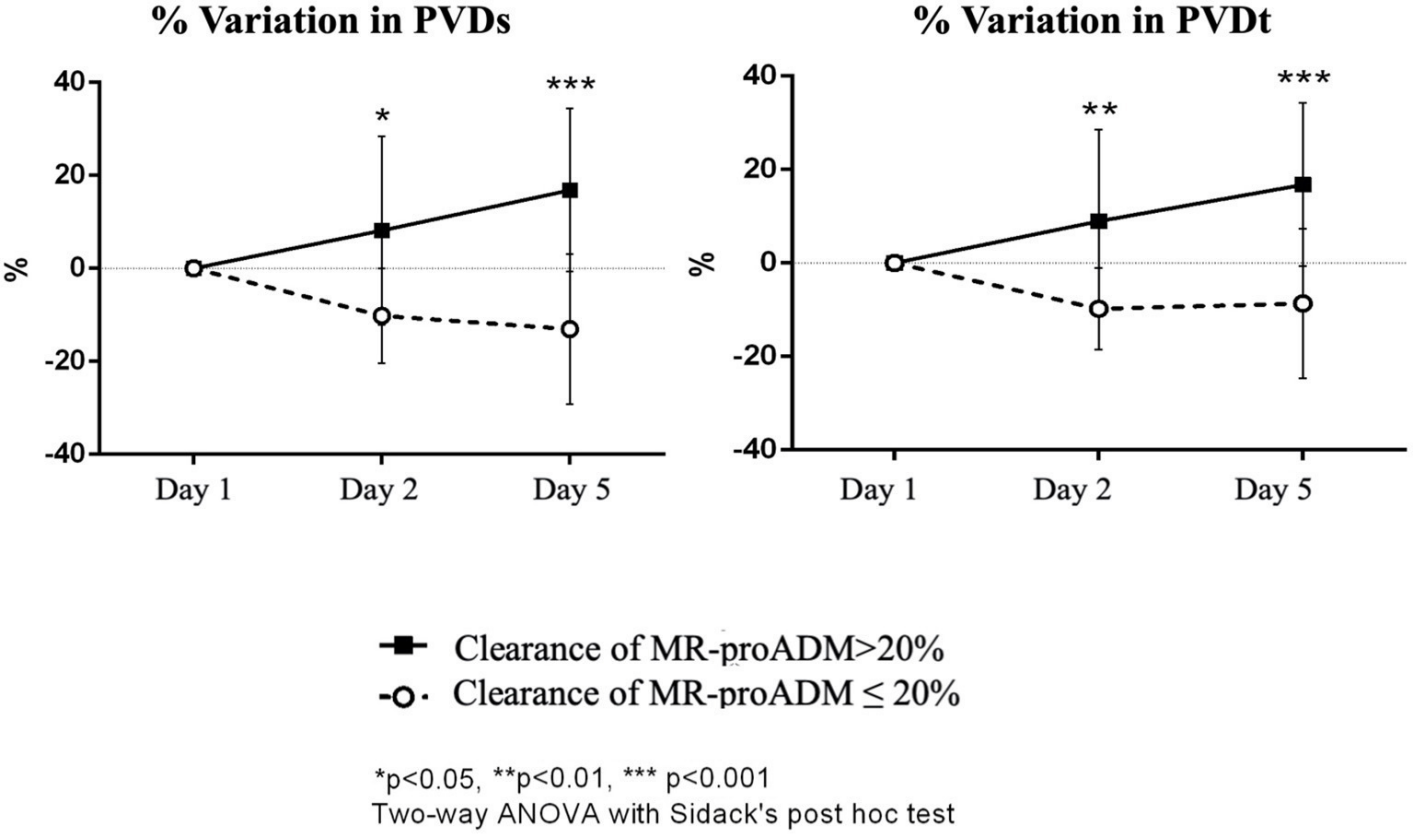
Percentual variation of perfused vessel density of small vessels (PVDs) and PVD of total vessels (PVDt) from baseline in the two groups of patients (clearance of MR-proADM inferior-to-equal or higher than 20%). The two-way ANOVA with the Sidack's *post-hoc* test. ^*^p < 0.05. ^**^p < 0.01. ^***^p < 0.001.

The two-way ANOVA for repeated measures (Box-Cox transformation) showed for PVDs a significant interaction between time and group (*p* = 0.004). The Sidack's test was not significant at any time points; however, there was a significant increase in PVDs at day 5 in the group of patients with clearance of MR-proADM > 20% (*p* = 0.024 vs. baseline) and a significant decrease in PVDs at day 5 in the group of patients with clearance of MR-proADM ≤ 20% (*p* = 0.035 vs. baseline). The two-way ANOVA performed on percentage of variation of PVDs from baseline found a significant interaction between time and group (*p* = 0.002) and a significant effect of group (*p* < 0.001). A difference between the two groups was evident at 24 h (*p* = 0.012) and at day 5 (*p* < 0.001). A significant increase in PVDs was found in the group of patients with clearance of MR-proADM > 20% at 5 days (*p* = 0.022 vs. baseline), while a significant decrease was found in patients with clearance of MR-proADM ≤ 20% at day 5 (*p* = 0.021 vs. baseline) (Figure 4).

For PVDt, a significant interaction was evidenced between time and group (*p* = 0.015); the Sidack's multiple comparisons test was not significant. PVDt tended to increase at day 5 in the group of patients with clearance of MR-proADM > 20% (*p* = 0.066 vs. baseline) and decrease at day 5 in the group of patients with clearance of MR-proADM ≤ 20% (*p* = 0.088 vs. baseline); however, the changes were not statistically significant. There was a significant interaction between time and group (*p* = 0.006) and a significant effect of group (*p* = 0.001) in the two-way ANOVA performed on percentage of variation of PVDt from baseline with a significant difference between the two groups at 24 h (*p* = 0.007) and at day 5 (*p* < 0.001). A significant increase in PVDt was only found in the group of patients with clearance of MR-proADM > 20% at 5 days (*p* = 0.019 vs. baseline) (Figure 4).

For PPVs and PPVt, no significant interaction between time and group and no significant effect of time were found. The Sidack's multiple comparisons test revealed no significant difference between the two groups at any time points. Moreover, there was no significant change over time in PPVs or PPVt in either group.

The test also showed a significant interaction between time and group for De Backer score (*p* = 0.022) and for TVDs (*p* = 0.018); the Sidack's test was not statistically significant.

No other correlation was evidenced between MR-proADM and the De Backer score or TVD of total and small vessels.

### Mid-Regional proADM and the SOFA Score

A clearance of MR-proADM ≤ 20% in the first 24 h of ICU stay discriminated a worsening of the SOFA score from T2 to T5 (AUC 0.938, CI 0.776–1, *p* = 0.025) with the median SOFA score at day 5 of 13 (9–15) in the group where MR-proADM clearance was lower than 20 vs. 8% (6–11) in the group with higher clearance.

### The SOFA Score and Microvascular Variables

The SOFA score was correlated to microvascular variables at admission in ICU only in septic shock patients with the Spearman's rho correlation coefficient for MFIs of −0.698 (*p* = 0.025) and for PPVs of −0.720 (*p* = 0.017).

At day 2, the SOFA score was weakly correlated to PPVs (the Spearman's rho correlation coefficient −0.459; *p* = 0.042) also in the general population. The correlation was stronger in the group with clearance of MR-proADM > 20% (the Spearman's rho correlation coefficient −0.893; *p* = 0.007) than in the group with clearance of MR-proADM ≤ 20% (the Spearman's rho correlation coefficient −0.663; *p* = 0.014). Similar correlation was evident at day 2 between the SOFA score and PPVt (the Spearman's rho correlation coefficient −0.461; *p* = 0.041 (−0.883, *p* = 0.08 in the group with clearance of MR-proADM > 20% and −0.682, *p* = 0.01 for the group with clearance of MR-proADM ≤ 20%, respectively).

The SOFA score was not correlated to microvascular variables at day 5.

## Discussion

In this small-scale preliminary study, we consecutively recruited 20 adult patients admitted in ICU with or for infection, sepsis, or septic shock. We monitored them for 5 consecutive days, analyzing the plasmatic levels of MR-proADM, the main clinical parameters, and the scores of severity. We evaluated the sublingual microcirculation to understand if MR-proADM, as biomarker of organ failure and of ADM-activity on the endothelium, could be associated with alterations of the variables of microcirculation and in particular to the MFI at admission in ICU. We calculated the clearance of MR-proADM over the first 24 h of recruitment and we compared it to the evolution over time of microvascular variables and of the SOFA score.

Mid-regional proADM at recruitment (and admission in ICU) tented to be higher in septic shock patients, than in septic and infected ones, but the difference was not statistically significant. The first 24 h clearance showed relation with the SOFA score. Although MR-proADM was not statistically related to the MFI at admission in ICU, the reduction of plasmatic levels of MR-proADM in the first 24 h of intensive care treatment was associated with an improvement of the MFI that was more evident than in patients with reduced or no clearance of the marker. Patients in which MR-proADM cleared showed a substantial stability of the MFI toward the first 5 days and an improvement in the SOFA score, while the opposite group suffered a deterioration of sublingual microcirculation in terms of the MFI and showed the statistically higher SOFA score at day 5. Similar evolution was evident for other parameters of microcirculation, in particular PVD and PPV of small vessels that relate to the quality of the flow and to microvascular perfusion. The proportion of perfused vessels was inversely correlated to the SOFA score at day 2, strongly in patients with higher clearance of MR-proADM. The SOFA score was correlated to microvascular variables at admission in ICU, but just in the small group of patients with septic shock.

Sepsis and septic shock are characterized by increased endothelial permeability, endothelial barrier dysfunction, proinflammatory activation of endothelial cell, reduced deformability of red blood cells, alterations of the glycocalyx, and leukocyte adhesion and rolling; further mechanisms lead to microcirculatory flow disturbance in septic pattern (23–25). Damaged microcirculation is involved in the pathophysiology of organ dysfunction: it compromises tissue perfusion through the impairment of both the diffusion (reduced and heterogeneous capillary density) and convection (altered capillary flow) of oxygen and nutrients; the altered blood flow furtherly acts as trigger to tissue inflammation (23–25).

In this situation, as in further inflammatory diseases, ADM increases and MR-proADM parallelly to ADM (10, 23). The increase of ADM participates to stabilize the endothelial barrier and to optimize the junctional integrity; it protects endothelial cells and the microcirculation (9, 10, 26–29). This effect was demonstrated on endothelial cells from different vascular beds (lung pulmonary artery, umbilical vein, and brain) in *ex-vivo* and *in-vivo* models and on the ileal microcirculation of experimental rat model of *Staphylococcus aureus* alpha-toxin-induced sepsis (13, 26, 29). The relevance of ADM on the integrity of the microvasculature in sepsis is the trigger for this study.

In this study, the single value of MR-proADM was not correlated to microvascular variables at admission in ICU, but the 24 h clearance of MR-proADM was correlated with the quality of the microvascular perfusion, the density of microcirculation, and with the severity of organ dysfunction.

From the results of this study, we could hypothesize that patients who showed enhanced clearance of MR-proADM and where organ dysfunction and microcirculation both improved could be considered as patients who controlled the inflammatory source that triggered production of ADM (ratio 1:1 with MR-proADM) and in which the endothelial damage of microcirculation resolved (or was more controlled). The opposite result in the group where MR-proADM cleared more slowly may indicate persistence of synthesis and release of ADM due to an active organ dysfunction and also of microvascular dysfunction.

Full-scale prospective studies will be needed to confirm the hypothesis, but it would be consistent with previous studies and in particular with the study of Valenzuela-Sánchez et al. (19), where they reported that ongoing MR-proADM levels at 48 h following admission in ICU and clearance of the marker on day 5 following admission helped to determine unfavorable evolution in 104 patients with severe sepsis.

As a pilot study on a small number of patients, our investigation cannot provide any conclusive answer on the correlation between MR-proADM and the microvascular and organ perfusion for several and important limitations. As the estimation of the sample size was aimed to the primary objective of this study (correlation of the single value of MR-proADM and the MFI at admission in ICU), the population could be statistically underpowered for the secondary analysis performed on the clearance of MR-proADM and the MFI, the SOFA score, and other microvascular parameters. The decision to choose an arbitrary cutoff of the 24 h clearance of MR-proADM could influence the results of this study and the use of a different cutoff could lead to different results. There is limited literature about the clearance of MR-proADM; none about the cutoff of clearance at 24 h and this threshold value requires further validation (8, 30, 31).

These factors should be carefully considered when extrapolating the data reported. The results could be also affected by the difference in the rate of disappearance of MR-proADM and the rate of changes in the microcirculation. Moreover, this study was intentionally designed to include a heterogeneous population of patients with wide different degrees of infection-related illness and, therefore, different expression of MR-proADM and degrees of microvascular alterations. The two groups analyzed showed baseline differences and this determinant influences the reliability of the results. Some of the patients enrolled presented sepsis related to viral origin in which MR-proADM could have performed within given limits, if considered just as single value (admission value) without examining a trend, although few studies suggest it as effective prognostic tool (8, 32) and the advanced average age of our population may also have affected the results and should be cautiously considered in the limitations of our data.

The microvascular damage in our population was, indeed, less severe of what we can expect in a population of septic and septic shock patients (in particular for the MFI and PPV), as we also included patients with infection, but without sepsis where the microvascular impairment is less studied and less homogeneous; most of the patients presented with an MFI <3 at admission in ICU and this result was consistent with our previous study, the MicroDAIMON study (33), where we reported an abnormal MFI on day 1 of admission in 20.6% of the patients and in 55.7% of cases during ICU stay; on the other hand, the median value of the MFI was still acceptable even in septic shock patients and the damage of the microcirculation was expressed heterogeneously among patients. The density of vessels (that is more strongly related to outcome in septic patients) was less impaired than expected, while the dominant alteration was the quality of the perfusion (6, 34).

For all the limits of this study explained, future full-scale studies should be performed in a more homogeneous population.

Although we suggest caution in reading our findings for the limitations that we already explained, we believe that our results may justify further research projects to evaluate if the trend of MR-proADM toward days could be able to relate to the evolution of microvascular dysfunction and of organ injury. If MR-proADM confirms the correlation found, it may play a clinical role as a biomarker in predicting the microvascular response to infections, sepsis, or septic shock. There are limited publications on the usefulness of MR-proADM in the field of critical care; we suggest this will be a field of interest (15, 32, 35–38).

## Conclusion

In our small cohort of adult patients admitted in ICU with infections, sepsis, and septic shock, MR-proADM and the MFI were not correlated at admission in ICU; however, a correlation existed between the first 24 h clearance of MR-proADM and the MFI and between the clearance of the molecule and other indices primarily connected with the quality of perfusion of the sublingual microcirculation. We believe that the relation between the expression of MR-proADM and the infection-related microvascular impairment merits further investigation: the clearance of MR-proADM could be a variable of interest in a comprehensive evaluation of this type of patients, but full-scale studies are needed to confirm our findings.

## Data Availability Statement

The raw data supporting the conclusions of this article will be made available by the authors, without undue reservation.

## Ethics Statement

The studies involving human participants were reviewed and approved by Comitato Etico Regione Marche - CERM; protocol number 212639, NCT03931967. The patients/participants provided their written informed consent to participate in this study.

## Author Contributions

RD: formal analysis, investigation, methodology, and writing. ED: investigation, methodology, and review and editing. CS, AC, EC, JM, VG, and EA: investigation. PG: investigation and analysis. MB and MM: analysis. AD: conceptualization, methodology, writing—review and editing, and project administration. All the authors read and approved the final manuscript.

## Conflict of Interest

The authors declare that the research was conducted in the absence of any commercial or financial relationships that could be construed as a potential conflict of interest.

## Publisher's Note

All claims expressed in this article are solely those of the authors and do not necessarily represent those of their affiliated organizations, or those of the publisher, the editors and the reviewers. Any product that may be evaluated in this article, or claim that may be made by its manufacturer, is not guaranteed or endorsed by the publisher.

## Supplementary Material

Supplementary Figure 1 Total vessel density (TVD), proportion of perfused small vessel (PVD), and PPV of small and total vessels, De Backer score in the two groups of patients (clearance of MR-proADM inferior-to-equal or higher than 20%). The Friedman test with the Dunn's *post-hoc* test. ^*^*p* < 0.05 for the Dunn's *post-hoc* test.
