# Supplementary figures and images for: Mid-Regional Proadrenomedullin (MR-proADM) and Microcirculation in Monitoring Organ Dysfunction of Critical Care Patients With Infection: A Prospective Observational Pilot Study
Source: Front Med (Lausanne). 2021 Nov 30;8:680244. doi: 10.3389/fmed.2021.680244 (PMC8669477; doi:10.3389/fmed.2021.680244)

TVDt

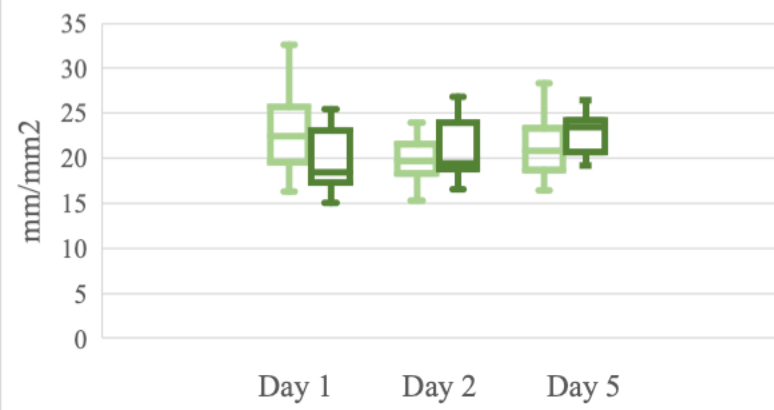

TVDs

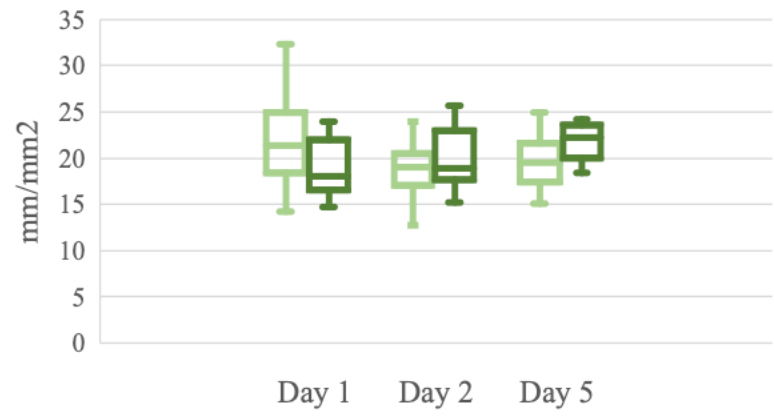

De Backer score

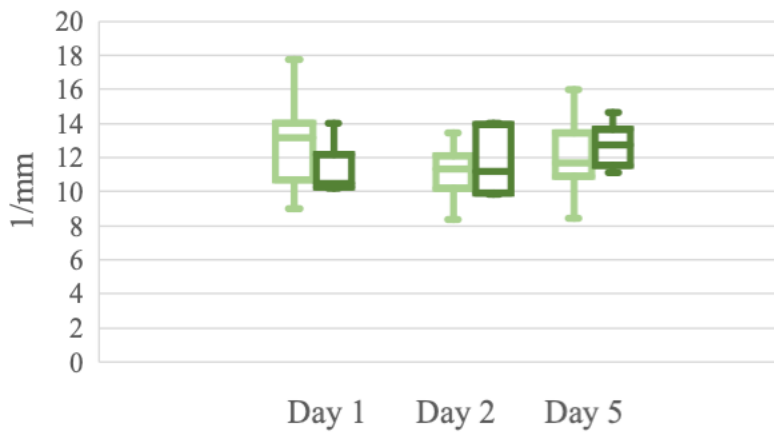

□ Clearance of MR-proADM  $\leq 20\%$   
■ Clearance of MR-proADM  $> 20\%$

PVDt

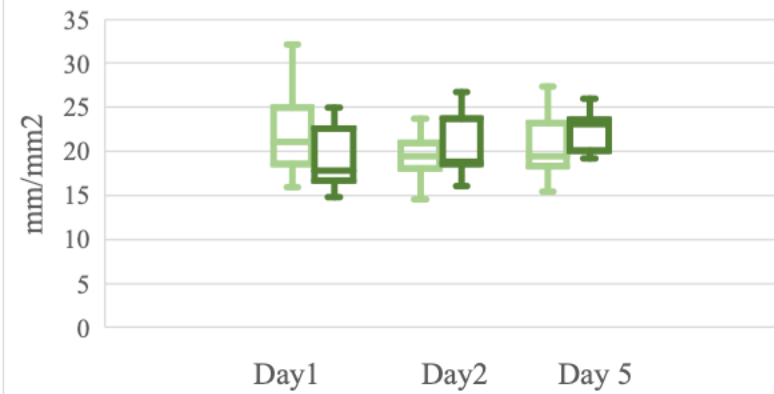

PVDs

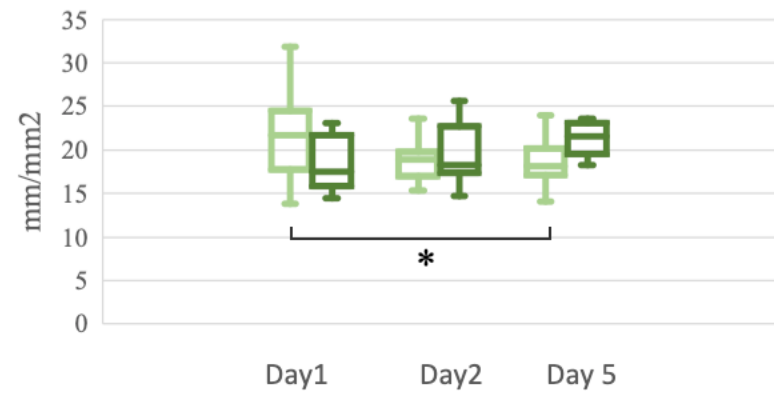

PPVt

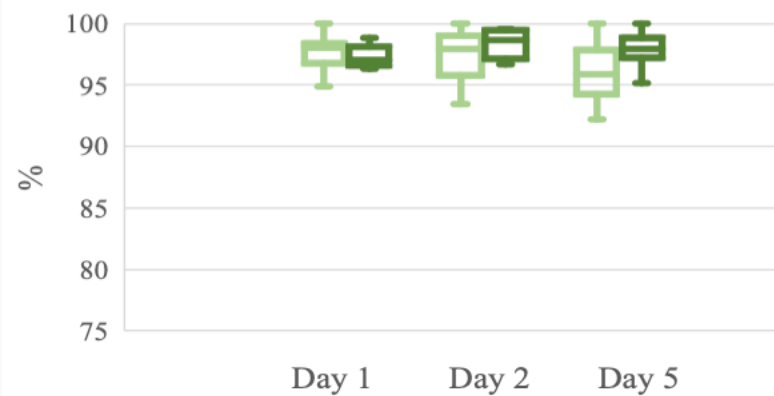

PPVs

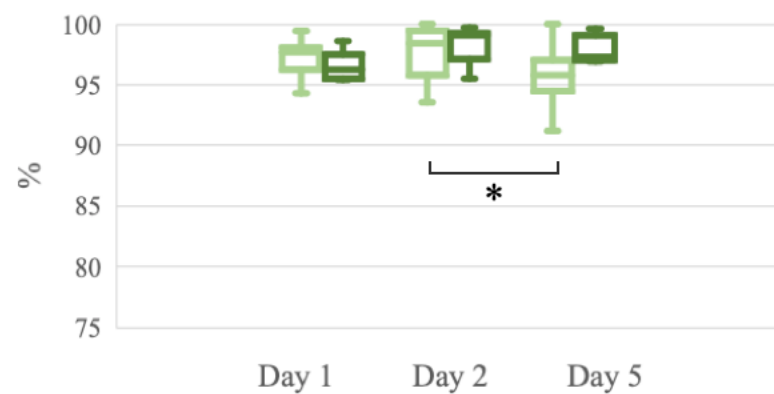

Supplement: Supplementary Figure 1 — Total vessel density (TVD), proportion of perfused small vessel (PVD), and PPV of small and total vessels, De Backer score in the two groups of patients (clearance of MR-proADM inferior-to-equal or higher than 20%). The Friedman test with the Dunn's post-hoc test. *p < 0.05 for the Dunn's post-hoc test. [file Image_1.pdf]
